# Tobacco-Smoking and Disease

**Published:** 1890-04

**Authors:** 


ARTICLE VIII.

### TOBACCO-SMOKING AND DISEASE.

There is a pretty widespread opinion among the laity that the use of tobacco serves to ward off the danger of contracting disease. Those who chew tobacco believe that this practice lessens the liability to caries of the teeth, and many who smoke consider smoking a fairly useful antiseptic measure. This popular opinion is probably due—as most such impressions are—to the impression made by numerous, though unsystematically observed, experiences. But of late the effect of tobacco-smoking has been studied carefully from a variety of standpoints, and largely because the custom has been attacked by moralists, who have pushed their line of attack into the domain of physiology and pathology. From deprecating the use of tobacco as selfish and unseemly, and as obnoxious to those who do not smoke, they have advanced to the assertion that it is pernicious to health and often dangerous to life.

Such assertions have drawn out many replies from medical men, who, as a class, have the habit of smoking; and many able papers have been written to maintain that temperate smoking is by no means injurious to men who are not unfitted for it by some peculiar weakness.

Further than this, at a recent meeting of the Vienna Doctoren Collegium a discussion arose in regard to the effect of tobacco-smoking upon the spread of certain diseases, especially diphtheria.

Dr. S. Hajek, of Vienna, prompted by experiments of Tassinari, of Pisa, on the destructive influence of tobacco-smoke on bacteria, reported some investigations of his own to determine the proportion of the cases of diphtheria among smokers and non-smokers. For this purpose he had collected data for the preceding three years, considering all individuals of the male sex upward of twenty-two years of age as smokers, which in Vienna is by no means a strained assumption. Among these, three thousand persons had had diphtheria during the period taken, and the proportion of the smokers to the non-smokers was, on an average, 1 to 2.8. Dr. Hajek directed attention to a statistical work by Professor Oser, bearing on this subject of the proportion of cases of diseases in men and in women in a certain epidemic of exanthematous typhus. Oser had observed that three times as many women as men were affected by the disease. The explanation of this difference was considered to be that men, in most cases, lived for only a few hours in insanitary dwellings, and thus became infected less easily than women. This difference ought also to be taken into account in collecting the data for diphtheria. Dr. Neudorfer, at the meeting referred to, called attention to the fact that a real destroyer of bacteria, pyridine, is present in tobacco-smoke; and Dr. Schiff added that in all bacteriological laboratories smoking was forbidden, as the smoke undoubtedly hindered the growth of the cultivated bacteria.

Putting all this together, we may conclude that there is probably some scientific ground for the popular belief that tobacco-smoking is of use as a preventive of disease. In saying this, we do not touch upon the æsthetic relations of the habit, but simply regard it from the scientific standpoint.—*Medical and Surgical Reporter.*

